# Quantifying the Impact of Winter Cover Crops on Sediment Export in Small Agricultural Watersheds and Beyond

**DOI:** 10.1007/s00267-026-02578-y

**Published:** 2026-08-01

**Authors:** Abagael N. Pruitt, Jennifer L. Tank, Shannon L. Speir, Ursula H. Mahl, Mohamed Aboelnour, Anna E. S. Vincent, Todd V. Royer

**Affiliations:** 1 Department of Biological Sciences, University of Notre Dame, Notre Dame, IN 46556 USA; 2 Environmental Change Initiative, University of Notre Dame, Notre Dame, IN 46556 USA; 3 O’Neill School of Public and Environmental Affairs, Indiana University, Bloomington, IN 47405 USA; 4 Present Address: Department of Environmental Toxicology, University of California, Davis, Davis, CA 95616 USA; 5 Present Address: Department of Crop, Soil, and Environmental Science, University of Arkansas, Fayetteville, AR 72701 USA; 6 Present Address: Center for Water Research, Northwestern University, Evanston, IL 60208 USA

**Keywords:** Conservation, Turbidity, Agriculture, Water quality

## Abstract

Agricultural land use increases the potential for soil erosion when the absence of vegetative cover during the fallow period makes fields vulnerable to runoff. Soil erosion can decrease crop yields and increase sedimentation in downstream water bodies. Agricultural conservation practices, such as the planting of winter cover crops, may protect soils and reduce erosion. To quantify the effect of cover crop use on field- and watershed-scale sediment export, we measured turbidity from tile drains and monitored continuous turbidity at the outlets of two agricultural watersheds in Indiana, USA, over seven water years (Oct 2016–Oct 2022). The watersheds differed in cover crop coverage: Shatto Ditch Watershed (23–68% coverage) and Kirkpatrick Ditch Watershed (8–30% coverage). We related turbidity to total suspended solids (TSS) using grab samples, and generated a record of daily TSS loads. We found that, on average, TSS losses from tiles draining fields with cover crops were 26% lower than fields without cover crops at Shatto, and 65% lower at Kirkpatrick (paired Wilcoxon signed-rank test; *p* < 0.001 for both watersheds). Average annual TSS yield from Shatto (159 kg/ha/yr) was 30% less than Kirkpatrick (226 kg/ha/yr). We also explored how TSS yields in these two small agricultural watersheds compared to TSS yields in river basins in the state of Indiana, and found that Kirkpatrick yields were significantly higher than the three major basins (ANOVA; *p* < 0.05), but Shatto TSS yields were not (ANOVA; *p* > 0.1). Overall, cover crops may reduce sediment loss from agricultural landscapes, especially during high-risk periods, helping protect sensitive downstream waters.

## Introduction

Agriculture provides food for a growing population (Viana et al. 2022; Milner and Boldsen 2023), and the successful preservation of topsoil is critical, as it holds nutrients, stores carbon, and can play an important role in maintaining soil fertility and crop yields (Silver et al. 2021; Koudahe et al. 2022). However, the production of row crops (e.g., corn and soybeans) can alter soil physical properties through intensive practices like conventional tillage, where soil is fully overturned after cash crop harvest (Glendell and Brazier 2014), or allowing land to remain bare during the fallow period between cash crop harvest in the fall and planting in the spring. For example, these practices can increase soil compaction, reduce water infiltration capacity, and increase the potential for erosion (Montgomery 2007; Du et al. 2022), while also reducing soil nutrients and compromising carbon storage (Blanco-Canqui and Ruis 2020; Lv et al. 2023). The potential for soil erosion is especially apparent during winter snowmelt and spring storms that occur during the fallow period when ground is bare, while the presence of subsurface tile drainage in poorly drained fields can increase or decrease particulate losses from fields by preventing ponding and overland flow (Williams et al. 2016). As such, overland flow during storms, as well as the soils lost via preferential flow through soil macropores into tile drains, can increase sediment loads to nearby waterways (Streeter et al. 2018; Guertault and Fox 2020; Vale and Dymond 2020). This has led to sedimentation in downstream waterways and reservoirs (O’Neal, 2005), which degrades freshwater systems in agricultural landscapes, compromises surface water supplies, and makes flood control less successful (Schleiss et al. 2016). Up to 1% of global reservoir storage is lost annually to sedimentation (Basson 2009). Finally, losses of carbon- and nutrient-rich topsoil can decrease crop yields (Basche et al. 2016). Thus, agricultural conservation practices that reduce soil loss from fields are needed to protect both farmers from financial losses associated with erosion (O’Neal et al. 2005; Chen et al. 2022), as well as water quality in waterways in agricultural landscapes (Blanco-Canqui 2018).

The planting of winter cover crops during the fallow season can provide green cover on bare fields, potentially retaining topsoil, by reducing water erosion during storms and snow melt (Singh et al. 2018; Chen et al. 2022). In the Midwestern United States, farmers are increasingly planting cover crops, with cover crop adoption in 2021 4X that of 2011 (Zhou et al. 2022). Many farmers plant cover crops such as cereal or annual ryegrass, as their extensive root systems help prevent soil compaction and can improve soil structure (Wong et al. 2024). In addition to improved soil physical properties, cover crops can improve soil nutrients (Chistopher et al. 2021) thereby improving water quality through reduced nitrogen and phosphorus losses to adjacent waterways (Hanrahan et al. 2021a; Speir et al. 2022; Thapa et al. 2024). Despite their potential benefits, the use of cover crops remains relatively low in the United States but is increasing (Zhou et al. 2022) and there are few studies that quantify cover crop effects on sediment loss (Blanco-Canqui et al. 2015). Additionally, because agricultural soils vary widely in their physical and chemical characteristics, the effectiveness of these practices in minimizing soil erosion may depend on local conditions, making them more effective on some fields than others (Blanco-Canqui et al. 2015; Lucas et al. 2022). Thus, a deeper understanding about the effects of cover crops on the loss of total suspended solids (TSS), at the field- and watershed-scale is needed, and could be used to promote this conservation practice among farmers and improve adoption rates.

To fully assess the impact of cover crops on TSS export, it is essential to consider their effects not just locally, but also on sediment export at the scale of larger systems. Much of the past research on cover crops and water quality has focused on small streams, ditches, and experimental systems (Hanrahan et al. 2021a; Badon et al. 2022; Kaur et al. 2024), but their effect on sediment export to downstream systems, and how these sediment yields from small watersheds compare to those from larger river basins, remains understudied (Blanco-Canqui et al. 2015). One study found that cover crops reduced sediment export by 58% in the lower Mississippi River Basin using the Agricultural Policy Environmental eXtender (APEX) model (Thapa et al. 2025). In another study, modeling results using the Soil and Water Assessment Tool (SWAT) found that cover crops reduced TSS export by up to 30% in the St. Joseph River basin (Aboelnour et al. 2025b). Many large river basins are of significant concern due to elevated sediment export, given that they are tributaries to large downstream bodies of water, such as the Laurentian Great Lakes. Placing small agricultural watersheds with high cover crop use into the context of river basin exports, especially for systems of concern in key agricultural states like Indiana, will help improve our understanding of the benefits of cover crop adoption at multiple spatial scales. This context will also be critical as climate change continues to increase the frequency and intensity of storm events in the Midwest (Byun et al. 2019; Hamlet et al. 2020).

Winter cover crops are often promoted as a strategy to reduce sediment loss, but there is limited understanding of whether field-scale benefits are detectable downstream at the watershed- or large river basin-scales. Here, we documented the effect of winter cover crops on field- and watershed-scale TSS loss in two small agricultural watersheds, and compared the TSS yields of small watersheds to that of larger river basins in Indiana. We asked: How does vegetative land cover in the fallow period impact stream sediment loading and export, and are these changes detectable at field-, watershed-, and larger river basin scales? Plant roots stabilize soil and reduce susceptibility to erosion (Wong et al. 2024), and this is the presumed mechanism by which cover crops reduce TSS loads in receiving waterways. We hypothesized that TSS losses will be lower from tile drains draining fields with cover crops than fields without, because plant root systems stabilize the soil. We also predicted that TSS yields (mass loss per unit area) will be higher in a watershed with little cover crop coverage compared to a watershed with more cover crop planting because there is less plant biomass to retain soil on the landscape. Larger rivers often have higher particle retention and settling due to their increased depth, as well as the presence of biota (e.g., freshwater mussels) that filter sediment from the water column (Vaughn 2018). Additionally, larger rivers typically have slower velocities, lower gradients, and greater depths, which can reduce turbulence and limit sediment resuspension (Dubuis and De Cesare 2023). Therefore, we hypothesized that TSS yields will be lower from larger river basins compared to the small watersheds.

## Materials and Methods

### Study Sites

**Fig. 1:**
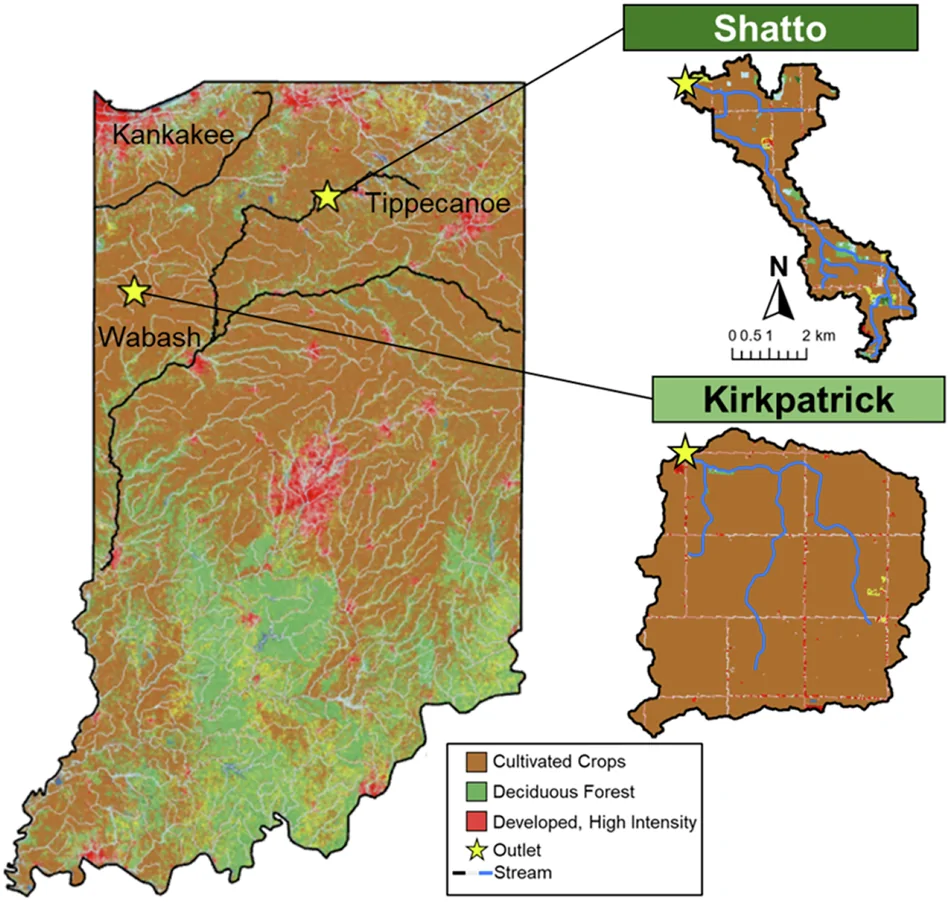
Map of study watersheds in Indiana, USA, including Shatto Ditch Watershed (top) and Kirkpatrick Ditch Watershed (bottom), where the stars represent the watershed outlet. The Kankakee, Tippecanoe, and Wabash rivers are denoted in black on the map of Indiana, and map colors represent land use (USGS 2019)

We sampled two small agricultural watersheds as part of the larger Indiana Watershed Initiative (Hanrahan et al. 2018; Trentman et al. 2020a; Speir et al. 2022; Vincent et al. 2025): Shatto Ditch Watershed (Kosciusko County, IN) and Kirkpatrick Ditch Watershed (Jasper County, IN), which are dominated by row crop agriculture (>85%; soybean-corn rotation; Hanrahan et al. 2018) and include subsurface tile drainage and channelized ditches (Fig. 1). The two watersheds differ in size and drain into different Indiana River basins; Kirkpatrick drains 2630 ha into the Iroquois River, while Shatto drains 1333 ha into the Tippecanoe River. Soils in Shatto are primarily alfisols and mollisols, and are sandy loam in texture, while in Kirkpatrick, soils are mostly mollisols, with a silty clay loam texture (Christopher et al. 2021). Sediment substrates in the waterways that drain the watersheds are also different; Shatto sediments are primarily fine benthic organic matter (FBOM) and sand, whereas Kirkpatrick sediments are dominated by gravel, with some additional FBOM (Roley et al. 2012; JL Tank, unpublished data). Both watersheds are drained extensively by subsurface tiles, and overland flow was rare during the study period. Additionally, watershed slopes are similar at ~0.3%. Tile drainage intensity is comparable between watersheds, and while surface intakes are present in the watersheds, their influence was likely negligible given the limited occurrence of overland flow. Continuous discharge (Q) was measured at United States Geological Survey gages at the outlet of each watershed (Shatto: #03331224; Kirkpatrick: #05524546; U.S. Geological Survey 2025). In general, mean Q was generally higher in Kirkpatrick than in Shatto.

Farmers in both watersheds use a range of tillage practices, including conventional tillage, or conservation tillage like mulch or reduced till, and no tillage (Speir et al. 2022), and the two watersheds also differed in cover crop coverage over the study period (Table 1; Fig. S1). We determined cover crop coverage in each watershed by recording the presence or absence of cover crops in each field during each year. In Kirkpatrick, the cover crop coverage ranged from 8 to 30% (average = 17%) from water years 2016–2022, while in Shatto, cover crops ranged from 23 to 68% (average = 50%). At Kirkpatrick, we documented that farmers planted a variety of cover crop species, but cover crops were dominated by cereal rye (*Secale cereale*) and annual rye (*Lolium multiflorum*), but also some oats (*Avena sativa*) and radish (*Raphanus sativus*) were planted. At Shatto, farmers generally planted annual or cereal ryegrass. Previous studies from our group have documented how cover crops impact soil health properties (Christopher et al. 2021), as well as the losses of nitrate (NO_3_^-^-N), soluble reactive phosphorus (SRP), and ammonium (NH_4_^+^-N) from tile drains and at the watershed scale (Hanrahan et al. 2018; Trentman et al. 2020b; Speir et al. 2022; Vincent et al. 2025). As part of the Indiana Watershed Initiative, we provided farmers with financial incentives to plant winter cover crops from water year 2016–2019. After incentives ended, cover crop coverage (as % of croppable acres) decreased (Table 1). For context, the average cover crop use in Indiana remains steady at ~10% of cropland (ISDA, 2021), so even with the declines after water year 2019, the two study watersheds had greater than average coverage during the study period.

**Table 1:** Watershed size in hectares (ha) and cover crop coverage (%) in the five Indiana study watersheds

Watershed	Watershed Size (ha)	2016	2017	2018	2019	2020	2021	2022
Shatto	1333	68	62	62	68	23	35	30
Kirkpatrick	2660	23	24	13	32	12	11	8
Kankakee	493300	11	10	12	12	20	20	19
Tippecanoe	491300	8	10	7	5	12	12	10
Wabash	11608400	9	10	8	8	10	10	10

### Sample Collection and Analysis

To monitor suspended sediments, we deployed Hach Hydrolab MS5 sondes (Hach Hydromet, Loveland, CO) at the outlets of both watersheds to collect continuous water-column turbidity (in Nephelometric Turbidity Units; NTU) every 30 min from water year 2016-2022. We also collected grab samples for turbidity every two weeks from the majority of tile drains and along the waterway in each watershed; we measured turbidity in these samples using a handheld turbidimeter (Hach 2100Q; Hach, Loveland, CO). We sampled *n* = 17 tile drains and *n* = 9 stream sites in Shatto, and *n* = 16 tile drains and *n* = 6 stream sites in Kirkpatrick; we include only tile drains that drain individual fields rather than any larger county drains (Speir et al. 2022). Each year, we classified each drain as cover cropped or not cover cropped (no cover crop) based on the coverage during the fallow period. At the time of sampling, we measured instantaneous Q (L s^−1^) from each tile drain using a bucket and stopwatch, but when Q was too high to use this method (>10 L s^−1^), we measured depth (*d*; in m), tile drain radius (*r*; in m), and velocity (*v*; in m s^−1^) using a Marsh McBirney Flo-Mate flow meter (Marsh-McBirney, Inc, Frederick, MD) and calculated Q using the following equations:

1 $$\theta ={\cos }^{-1}\left(1-\frac{d}{r}\right)$$

2 $${Q}_{{TD}}={r}^{2}\left(\theta -\cos \theta \sin \theta \right)\times\,v\,\times\,1000$$

where *Q*_*TD*_ represents tile drain *Q*, *d* is depth, *r* is tile drain radius, and *v* is velocity. We also note that we did not collect tile drain samples when tile drains were submerged during high flow events (<2X per year), nor when the tile drains were not flowing (i.e., dry) or frozen.

To measure TSS over the study period, we collected grab samples from the water column using 1 L Nalgene bottles (Thermo Fisher Scientific, Waltham, Massachusetts) from the outlets of both watersheds, consistently sampling the well-mixed water column from ~30 cm below the surface to collect suspended sediments not directly above the benthos or directly below the surface. We collected biweekly samples for ~1.5 years between 2021 and 2022 across a range of hydrologic conditions, including baseflow and stormflows, to capture variability in TSS concentrations. We returned to the laboratory and filtered the sample onto a pre-ashed and weighed Type A/E glass fiber filter. We dried the filter at 60 °C for 24 h, and reweighed for dry mass. To calculate TSS concentration (mg L^−1^), we subtracted the filter weight from the dried sample plus filter, then divided by the volume of water filtered (ASTM 2025).

### Data Analysis

**Fig. 2:**
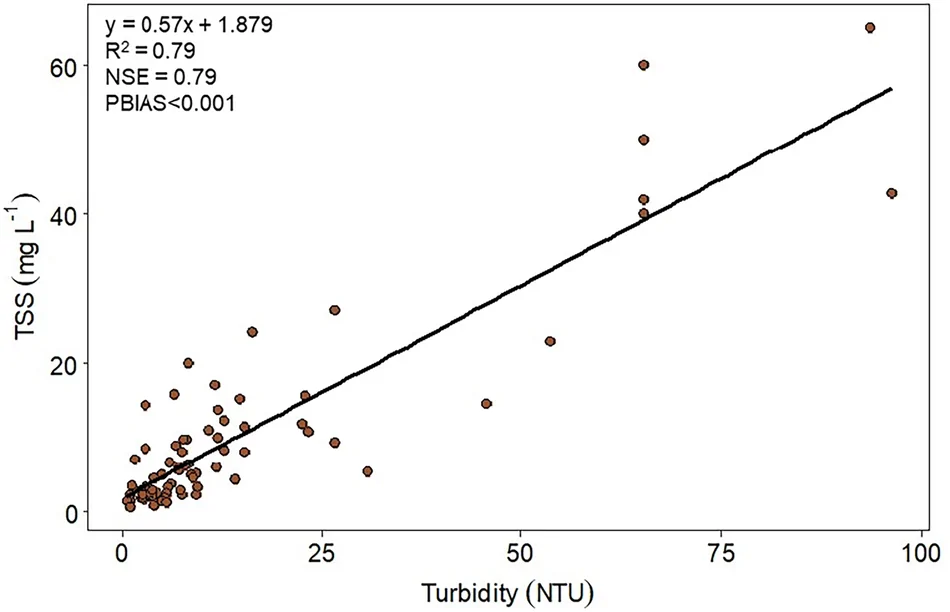
Turbidity (NTU)-total suspended solids (TSS) (mg/L) relationship created using 78 grab samples at Shatto and Kirkpatrick (watersheds combined)

To generate a Turbidity-TSS relationship (Fig. 2), we related continuous turbidity data to biweekly grab samples for TSS using linear regression (Helsel and Hirsch 2020), resulting in the following equation:

3 $${TSS}=\left(0.571\,x\,{Turbidity}\right)+1.879$$

We used a single, combined regression across both sites to maximize the sample size and ensure model stability, particularly given the similar range of turbidity values at each site. Preliminary analyses indicated that the turbidity–TSS relationships at the two sites were not significantly different (ANCOVA; *F*(1) = 6.7, *p* = 0.44). For tile drain TSS, we used equation 3 to estimate TSS concentration (mg L^−1^) for each tile drain sample on each sampling date, and we multiplied tile drain Q by TSS concentration to calculate instantaneous tile drain TSS loss rates (kg d^−1^). We also applied the TSS-turbidity relationship to the continuous record of sonde turbidity data, to generate a multiyear record of estimated daily TSS concentrations, using the average turbidity value for each day. For each day, we then calculated TSS load (kg d^−1^) by multiplying TSS concentration by Q and we interpolated missing data (e.g., due to sensor fouling; Shatto = 482/2190 days, 22%, Kirkpatrick= 986/2190 days, 45%) using the Loadflex modeling package in R (Appling et al. 2015) and the rectangular interpolation function. To directly compare between Shatto and Kirkpatrick, we divided TSS load estimates by watershed area to calculate watershed TSS yield (kg ha^−1^ d^−1^).

To understand how flow impacted TSS yields, we performed a flow duration analysis to determine the proportion of TSS lost at different flow categories. Following US EPA (2007), we ranked all days in the study period from lowest to highest Q for Shatto and Kirkpatrick separately, then categorized each day as low flow (<60th percentile of flow), medium flow (60–90th percentile of flow), or high flow (>90th percentile of flow; i.e., storms).

As a comparative tool, and to analyze cover crop effects at coverages that exceeded actual adoption rates, we also estimated Winter and Spring TSS yields from Shatto and Kirkpatrick using the Soil and Water Assessment Tool (SWAT; Neitsch et al. 2009) at 0% cover crop coverage and at 100% cover crop coverage using the method described in Aboelnour et al. (2025b) and Hamlet et al. (2023). Briefly, we used the ArcSWAT interface, an ArcGIS-based graphical user interface (version 2012, ArcGIS 10.8), to facilitate watershed delineation, HRU definition, and the integration of spatial datasets required for model setup. 100% cover crop adoption is not representative of current conditions and may overestimate achievable reductions. However, we intentionally used the 100% scenario as a theoretical upper bound, meant to bracket observed adoption levels and explore potential watershed-scale responses under idealized conditions with some uncertainty from extrapolating outside the observed range of cover crop coverage. We used ArcSWAT to establish the model configurations for each watershed, then calibrated and validated the model using daily Q data from the two watershed USGS gauges. We also used the TSS data that we generated from each watershed at varying cover crop coverages (from 8-68%) to further calibrate the model. We represented tile drainage and channelized ditches using SWAT’s standard subsurface drainage and channel routing parameters appropriate for intensively tiled Midwestern agricultural systems. For timing of cover crop management, we set cover crop planting and termination dates as October 21 and May 5 of each year, respectively, reflecting operations typical of this region (personal observation). We performed four iterations of 2000 simulations using the Sequential Uncertainty Fitting program algorithm version 2 (SUFI-2) of the SWAT-CUP interface (Abbaspour 2011) to estimate monthly TSS loads under 0% cover crop coverage and 100% cover crop coverage (all annual ryegrass) for each watershed.

Finally, to compare TSS yields from these two small agricultural watersheds to major larger river basins in Indiana, we used publicly available TSS load data via the Purdue lthia Water Quality Trending Tool (https://lthia.agriculture.purdue.edu/trending/). This tool allows users to visualize water quality data, including TSS loads, from 1992 to present day for multiple Indiana rivers (Harbor 1994; Liu et al. 2015), which were calculated using adjusted maximum likelihood estimation (AMLE) in LOADEST (Runkel et al. 2004), with streamflow and TSS concentrations as inputs to the model. We used data from the Purdue trending tool to estimate annual TSS yields from the water year 2016–2022 study period from the Tippecanoe River, Kankakee River, and Wabash River basins (Fig. 1), which vary in drainage size and cover crop coverage (Fig. 1; Table 1).

### Statistical Analysis

To meet the assumptions of parametric statistics, we tested all collected data using a Levene’s test and a Shapiro-Wilk test for normality (*p* > 0.05). All data were normally distributed, except annual TSS yields, which we normalized using a logarithmic transformation. We averaged tile drain losses by field treatment for each sampling day, and performed a paired Wilcoxon signed-rank test to compare cover crop vs. no cover crop tile losses during winter and spring in each watershed. We also used the paired Wilcoxon signed-rank test to compare the effects of watershed (Shatto vs. Kirkpatrick) on annual TSS yields, with years treated as pairs. We used a one-way analysis of variance (ANOVA) to compare differences in watershed and river basin (Shatto, Kirkpatrick, Tippecanoe, Kankakee, and Wabash Rivers) annual TSS yields. We also used Pearson correlation (r) to relate TSS yields and SWAT-modeled reductions (%) in yields with cover crops to total water year precipitation. We used an alpha level of 0.05 as a significance criterion for our analyses, and following a significant difference, we used a Tukey’s HSD post-hoc test to identify differences among treatments. We completed all statistical analyses in R version 4.1.3 (R Development Core Team, 2016).

## Results

### TSS Losses from Fields With and Without Cover Crops

**Fig. 3:**
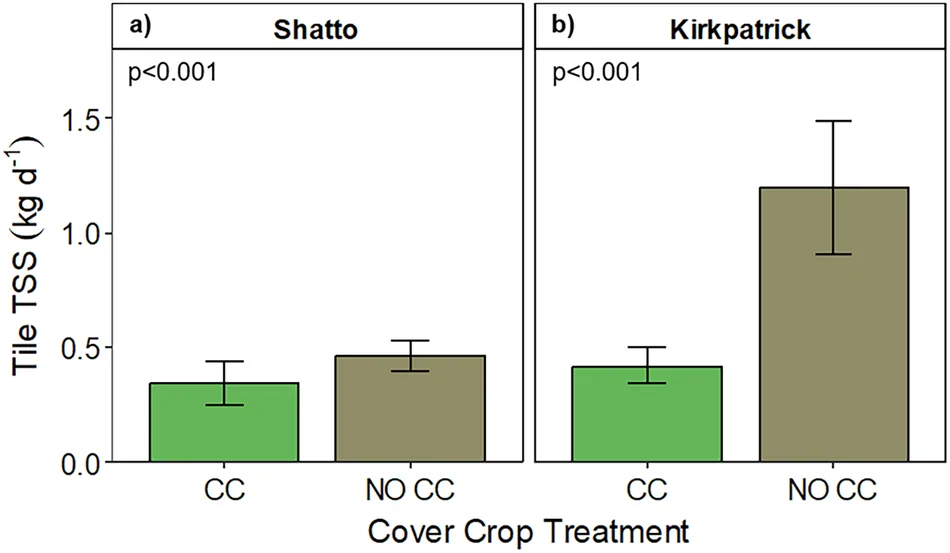
Mean ± SE winter and spring total suspended solids (TSS) loss in kg d^−1^ at Shatto (**a**) and Kirkpatrick (**b**) from tiles draining cover crop (CC) and no cover crop (NO CC) treatment fields

To understand how cover crops influenced TSS losses at the edge-of-field-scale, we compared TSS loss from tile drains draining fields with and without cover crops in winter and spring (Fig. 3). At Shatto (*n* = 586 samples, 232 paired samples), TSS losses from tile drains were 26% lower from tiles draining cover crop fields compared to no cover crop drains (Fig. 3a; paired Wilcoxon signed-rank test; *p* < 0.001). At Kirkpatrick (*n* = 263 samples, 43 paired samples), TSS losses from tiles draining cover crop fields were 65% lower than no cover crop tile losses (Fig. 3b; paired Wilcoxon signed-rank test; *p* < 0.001).

### Watershed-Scale TSS Yield

**Fig. 4:**
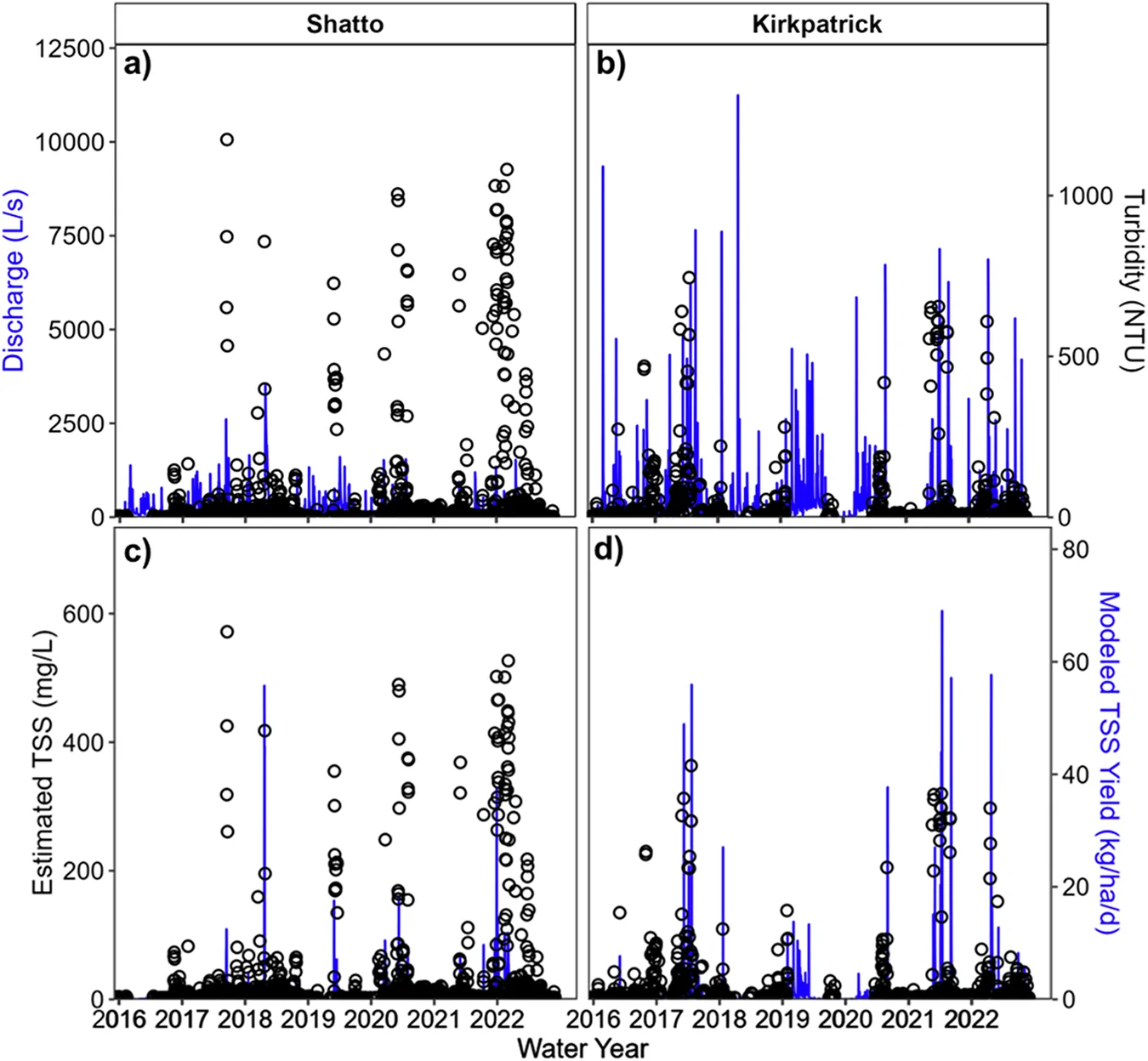
Time series of daily discharge (Q; L/s) and turbidity (NTU) at Shatto (**a**) and Kirkpatrick (**b**), and estimated total suspended solids (TSS) concentration (mg/L) and modeled TSS yield (kg/ha/d) at Shatto (**c**) and Kirkpatrick (**d**) from water year 2016 to 2022

We estimated daily TSS yields from water year 2016-2022 for both watersheds using daily Q, turbidity, estimated TSS concentrations, and modeled TSS yields (Fig. 4). In Shatto, daily *Q* ranged from 0.6 to 3540 L s^−1^ (mean = 186; Fig. 4a), and mean Q was ~2X lower than in Kirkpatrick (mean = 315; range = 0.3–11246; Fig. 4b). Daily turbidity in Shatto ranged from 0.4 to 1174 NTU (Fig. 4a), and mean NTU was ~7X lower than in Kirkpatrick (0.4-745 NTU; Fig. 4b). We estimated the range in daily TSS concentrations at Shatto to be from 2.3 to 572 mg L^−1^ (Fig. 4c) which was slightly higher than in Kirkpatrick (range 2.3–465 mg L^−1^; Fig. 4d). Daily TSS yields were variable in both Shatto (0.0002–56 kg ha^−1^ d^−1^; Fig. 4c) and Kirkpatrick (0.0004–69 kg ha^-1^ d^−1^; Fig. 4d).

**Fig. 5:**
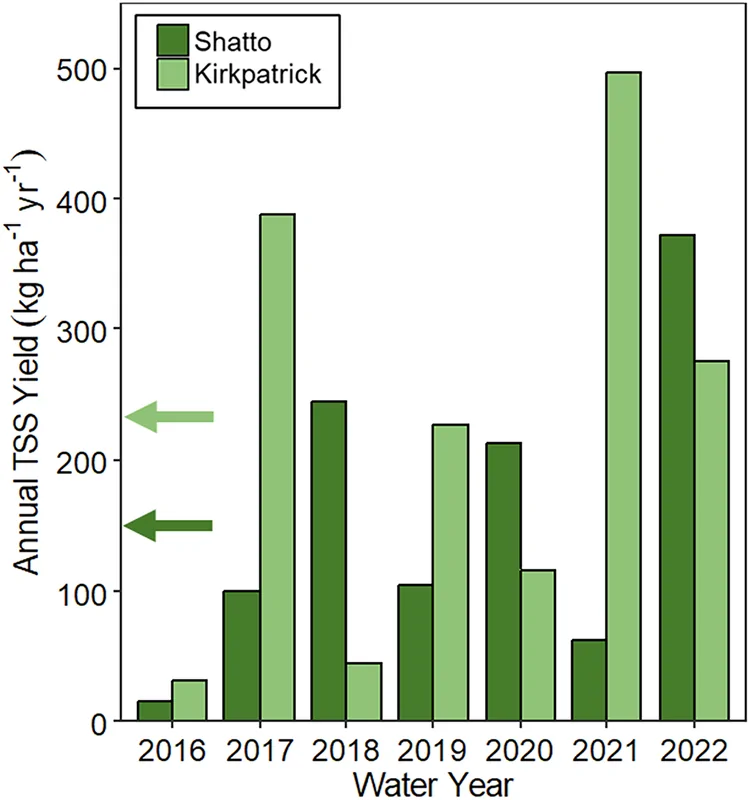
Annual total suspended solids (TSS) yields (kg/ha/yr) from Shatto and Kirkpatrick from water year 2016 to 2022. Arrows represent the overall mean of the annual TSS yields for each watershed

To understand how Shatto and Kirkpatrick annual yields varied over time, we summed the daily yields for each watershed for each water year (2016–2022; Fig. 5). For both watersheds, annual TSS yield was variable among years. At Shatto, annual yields ranged from 15-373 kg ha^−1^ yr^−1^, while at Kirkpatrick annual TSS yields were 32–497 kg ha^−1^ yr^−1^. Given the interannual variation, however, these means were not significantly different (paired Wilcoxon signed-rank test; *p* = 0.58). Finally, TSS yields were lower than in Kirkpatrick compared to Shatto for four out of seven study years (2016, 2017, 2019, 2021). In order to understand how precipitation might drive variation in yield, we related TSS yield to total water year precipitation (watersheds combined), but the correlation was not significant (*n* = 14 samples; Pearson *r* = −0.32, *p* = 0.26).

**Fig. 6:**
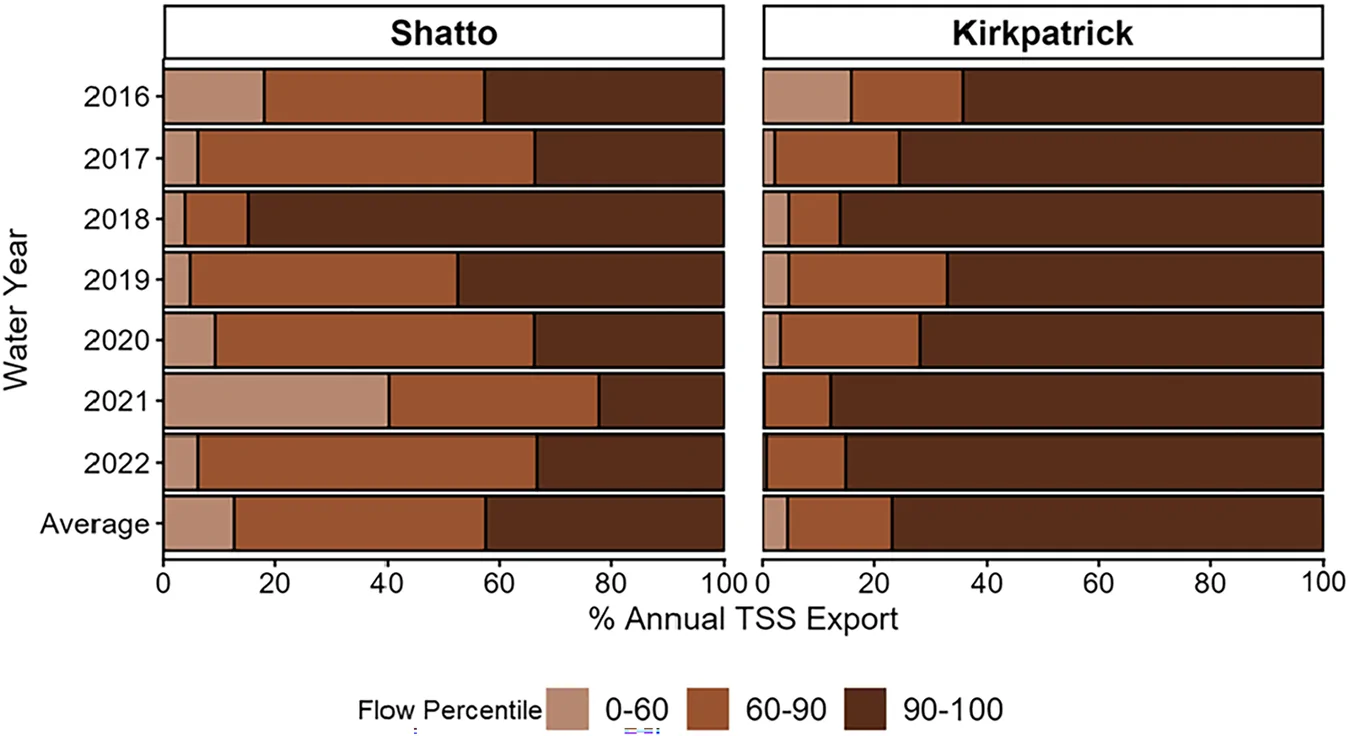
Flow duration analysis of total suspended solids (TSS) loads at Shatto (**left**) and Kirkpatrick (**right**) from water year 2016–2022. The bottom row represents an average of the seven water year period. Flow percentiles range from base flow (0–60% of flows; dark brown) to high flows (top 10% of flows; light brown)

To understand how TSS yields varied with flow, we performed a flow duration analysis to demonstrate that differences in TSS yields were more pronounced during storm flows (i.e., top 10%) relative to base flow conditions (Fig. 6). On average, for Shatto, highest flows (90th–100th percentile of flows) accounted for 43% of TSS yield (range 22–85%), while for Kirkpatrick, the highest flows averaged 77% of annual TSS yields (range 64–88%). The top 10% of flows produced the highest annual TSS yields in three of seven water years at Shatto, and were always the most prominent flow category for annual TSS yields at Kirkpatrick. On average, the medium flow category in Shatto made up 45% of TSS yield (range 11–61%), while in Kirkpatrick the average was 19% (range 9–28%). The medium flow category produced the highest TSS yields for four of seven water years at Shatto (2017, 2019, 2020, 2022), and never at Kirkpatrick. Conversely, the lowest flow category (0–60th percentile) at Shatto comprised 13% of TSS yield (range 4–40%), while at Kirkpatrick, low flow made up 5% of TSS yield (range 1–16%).

### Complete Winter Cover Crop Coverage Significantly Reduces TSS Yields

**Fig. 7:**
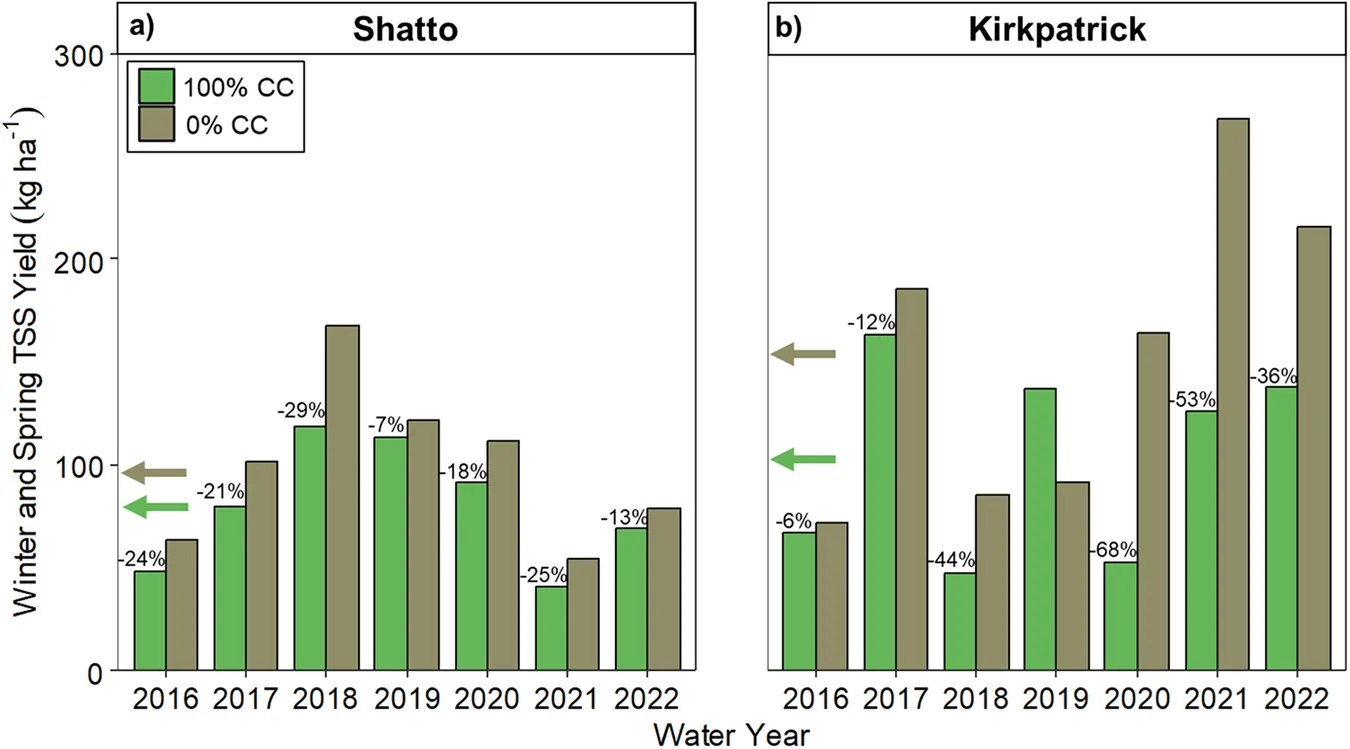
Comparison of Winter and Spring total suspended solids (TSS) yields (kg/ha) from SWAT modeling at Shatto (**a**) and Kirkpatrick (**b**) between 0% cover crop (CC) coverages and predicted yields with 100% of fields in the watersheds cover cropped. Arrows represent the overall mean of the Winter and Spring TSS yields for 100% CC and 0% CC for each watershed

We used a SWAT model to understand how maximum coverage of cover crops on all croppable acres could alter watershed-scale TSS yields (Fig. 7). We show that increasing cover crop coverage generally decreased watershed TSS yields, but that TSS yields also varied in Winter and Spring with and without cover crops. In Shatto, Winter and Spring TSS yields with 100% cover crop coverage ranged from 41 to 119 kg ha^−1^ compared to 54–167 kg ha^−1^ without cover crops (Fig. 7a), which suggests that cover crops could reduce TSS yields by 7–29%. In Kirkpatrick, 100% cover crop coverage reduced Winter and Spring TSS yields by 6–68%, with percent reductions in six out of seven water years, but these reductions were not related to total water year precipitation (*n* = 13 samples; Pearson *r* = −0.50, *p* = 0.08). Kirkpatrick TSS yields were very similar to Shatto when at 100% cover crop coverage; Winter and Spring TSS yields ranged from 48 to 163 kg ha^−1^, but they were much higher than Shatto without cover crops at 72–269 kg ha^−1^ (Fig. 7b).

### Comparing TSS Yields in Small Watershed vs River Basins

**Fig. 8:**
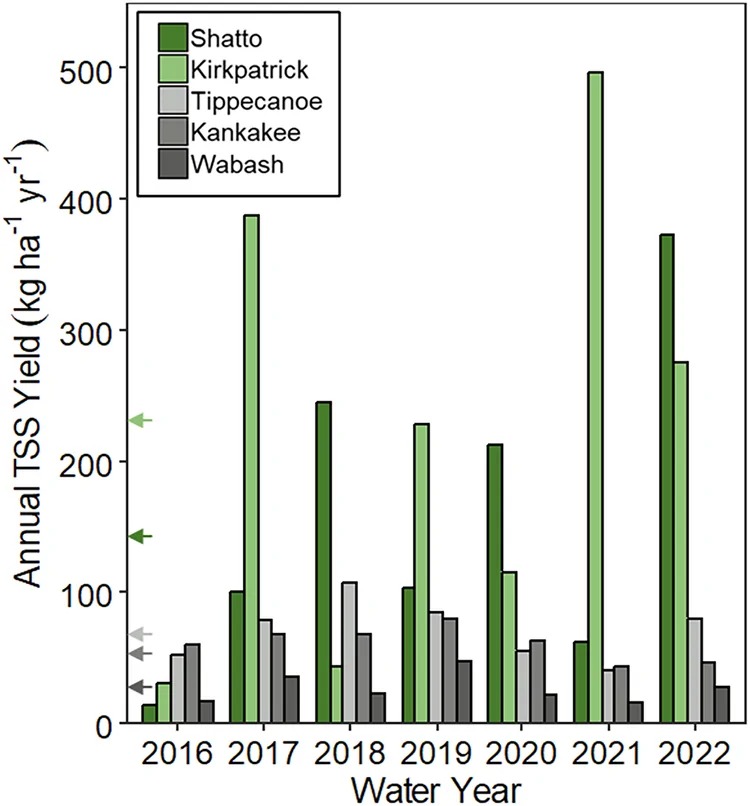
Annual total suspended solids (TSS) yields (kg/ha/yr) from Shatto, Kirkpatrick, Tippecanoe, Kankakee, and Wabash watersheds from water year 2016 to 2022. Arrows represent the grand mean of the annual TSS yields for each watershed

We compared TSS yields from the two small watersheds (Shatto and Kirkpatrick) with TSS yields from three nearby river basins in Indiana (Tippecanoe, Kankakee, Wabash Rivers) (Fig. 8) and found that annual TSS yields were higher in Shatto and Kirkpatrick than in the three large watersheds. We saw significant interannual variation among the three river basins, where annual TSS yield was highest for the Tippecanoe River, ranging from 40 to 109 kg ha^−1^ yr^−1^, followed by 44–80 kg ha^−1^ yr^−1^ for the Kankakee River, and was lowest for the largest basin (Wabash River) at 15–48 kg ha^−1^ yr^−1^. Average TSS yields were similar across the three river basins. We conducted this analysis on log-transformed TSS yields to meet model assumptions. Post hoc comparisons revealed that Kirkpatrick had higher TSS yields (ANOVA; *F*(4) = 4.9, *p* = 0.004), while TSS yields from Shatto were not significantly different from the rivers (ANOVA; *p* > 0.1 for all comparisons). In summary, while TSS yields varied across basins, the smaller watersheds, particularly Kirkpatrick, had higher sediment yields compared to the three larger basins.

## Discussion

### Cover Crops Reduce TSS Losses at the Field-Scale

Cover crops can influence the loss of TSS from tile drains through changes in soil structure (e.g., macropore development) but given the diversity of agricultural soils, their efficacy in reducing soil loss from fields may be site-specific (Blanco-Canqui et al. 2015; Lucas et al. 2022). We hypothesized that TSS losses will be lower from tile drains draining fields with cover crops because both aboveground and belowground plant biomass has a stabilizing influence on the soil. We found that TSS losses were significantly lower for tiles draining cover crops in both watersheds, with Kirkpatrick showing a larger average reduction in tile TSS losses, but more variability among sampling events. Previous studies by our group in these same watersheds have shown that cover crop planting reduced NO_3_^-^-N and NH_4_^+^-N losses in both watersheds, while the effect on SRP was variable (Trentman et al. 2020b; Hanrahan et al. 2021b; Speir et al. 2022; Vincent et al. 2025), and the results for soil N and P content generally mirrored the solute results (Christopher et al. 2021). Given the context-specific results for tile drain SRP losses and soil P reductions, the impact of cover crops on tile TSS loss may be similar. The contrasting tile-scale responses are consistent with known differences in soil texture, drainage behavior, and cover crop species composition. Root biomass and soil-specific sensitivity analyses may be useful for future studies to better understand the mechanistic differences between sites. Shatto soils are primarily sandy loams, while Kirkpatrick soils are dominated by silty clay loams, which differ in aggregation, erodibility, and sediment detachment potential, and cover crops may not have as large of an impact on soils which water and particles move through easily (Ritter et al. 1998; Thapa et al. 2024).

Some of the variability in TSS losses from Shatto and Kirkpatrick tile drains may be due to differences in cover crop selection, variable implementation across fields, and field-specific factors like drainage and soil characteristics. The type of plant selected for cover crops can also influence impacts on solute and particle transport (Kaur et al. 2024). For example, some producers in the Kirkpatrick watershed often planted oats and radish, which are killed by freezing temperatures during the winter, in contrast to ryegrass, which continues to grow until it is terminated via herbicide in the spring (Koudahe et al. 2022). In this study, we did not distinguish between overwintering and winter-kill cover crop species. Further research is needed to evaluate how different species may influence sediment loss and export. Through informal communications, we also found that many producers only planted cover crops on marginal or problematic fields, such as those with drainage issues (A. Pruitt, personal communication), which may have influenced TSS reductions. While cover crops are expected to increase soil macropore size (Lucas et al. 2022), we did not see an increase in tile flow (as Q) in either watershed, suggesting that increased porosity may have been offset by cover crop root stability (Lucas et al. 2022). Furthermore, variation in edge-of-field impacts makes detection at the watershed scale even more challenging (Hanrahan et al. 2021b; Speir et al. 2022).

### Cover Crops Reduce Watershed-Scale TSS Yields at High Flows

At the watershed scale, the published effects of cover crops on TSS yield have been variable, ranging from no effect on TSS (Badon et al. 2022) to a ~30% reduction in TSS export (Aboelnour et al. 2025b) with cover crops. We hypothesized that watershed TSS yields would be lower with higher cover crop coverage because there is more plant root structure underground, as well as aboveground plant biomass to retain soil on the land. Additionally, we predicted that the effects of cover crops on TSS yields may be more challenging to document at the watershed scale than at the field-scale, because there are more confounding factors, such as differences in land management throughout the watershed and climate-driven fluctuations in precipitation, and differences in topography and in-stream sediment dynamics. TSS yields showed significant interannual variation, which is typical of other studies in agricultural streams (Christensen et al. 2002; Meybeck et al. 2003; Landers and Sturm 2013). Yet we also found that TSS yields were similar between the two watersheds, despite differences between the two watersheds in cover crop coverage, soil type, and diversity of farm management practices (Christopher et al. 2021). These differences, along with annual weather variability, may obscure the effect of conservation practices at the watershed scale, despite their important role at the field-scale (Tomer and Locke 2011; Speir et al. 2022). We were unable to see a correlation between precipitation and TSS yields, suggesting site-specific variation in soils, management, and in-stream sediment dynamics were the primary reason for a lack of a watershed-scale effect. Storm intensity, duration, and antecedent conditions influence sediment export (Vale et al. 2020) and may further explain the lack of a relationship with precipitation. The lack of a measurable cover crop effect at the watershed scale has also been seen with dissolved nutrients, especially SRP, which exhibited patterns similar to TSS (Hanrahan et al. 2021a; Speir et al. 2022). This may be related to sorption patterns between SRP and sediments (Trentman et al. 2020a). With TSS, as studies have shown for dissolved nutrients and discharge, cover crops may have more of an impact at higher flows compared to base flows (Singh et al. 2018; Speir et al. 2021).

A significant portion of TSS export in streams often happens during high flows and storm events (Meybeck et al. 2003; Korup 2012; Ross et al. 2019), yet the effect of cover crops on storm-driven TSS export is understudied. Previous studies in these same watersheds have shown that 70-80% of annual dissolved NO_3_^−^-N losses and yields occur during the top 10% of flows, but cover crops can reduce this by 20% (Hanrahan et al. 2018; Speir et al. 2021). We did not directly compare storm magnitude and duration between watersheds, but total precipitation by water year was similar between the two sites, suggesting similar weather patterns for these two watersheds. When we examined the top 10% of flows for TSS yields, we found that more TSS was exported during those highest flows at Kirkpatrick compared to Shatto, perhaps suggesting that there was more resilience in Shatto soils and sediments to the largest storm events. The presence of cover crops during storms can delay the start of runoff, thus reducing sediment export and increasing resilience (Blanco-Canqui 2018; Thapa et al. 2024). While there is evidence that TSS are sourced from outside of the stream, where sources might include farm fields or hillslopes (Loperfido 2014; Sherriff et al. 2016), we must acknowledge that some portion of TSS can also come from within the stream (Vale et al. 2020), which would be challenging to distinguish using watershed-scale measurements of TSS yields during storms. In Shatto, the streambed sediments consist of finer particles, whereas Kirkpatrick is dominated by gravel. Despite this, higher flows contributed more to TSS yields at Kirkpatrick, likely due to increased mobilization of loosely stored sediment, differences in channel morphology, or variations in sediment supply from nearby fields and transport dynamics. At Shatto, there are constructed floodplains (i.e., two-stage ditch) over 4.1 miles (75%) of the stream, which slows water velocities during storms (Roley et al. 2012; Davis et al. 2015), and may be contributing to reduced TSS export during the highest flows. While the effect of cover crops on annual TSS yields at the watershed scale was not always detectable, we observed less TSS export in the top 10% of flows in Shatto, which had higher cover crop coverage compared to Kirkpatrick. Expanding cover crop coverage across watersheds could enhance these benefits and further reduce TSS yields over time.

Planting cover crops on 100% of fallow land could have major impacts on downstream water quality (Blanco-Canqui, 2018). We used a SWAT model as a synthesis and bounding tool to place the observed sediment reductions into a watershed-scale context that extends beyond currently achievable adoption levels, and found that cover crops reduced Winter and Spring TSS yields in all years at Shatto, and in most years at Kirkpatrick. One study in another nearby watershed showed reductions of TSS export by up to 30% when cover crops are used (Aboelnour et al. 2025b). Reductions here ranged from 6 to 68%, and in the year in Kirkpatrick in which we saw higher TSS losses with cover crops on the field, the difference between 100% cover crop and 0% cover crop scenarios was minimal. This shows the potential for cover crops to reduce soil erosion and subsequently reduce TSS yields when planted on more fields, thus accounting for more watershed land area. SWAT does not resolve individual tile–ditch connections or fine-scale drainage hydraulics, and the simulations are not meant to reproduce field-scale processes mechanistically. Subsurface tile drainage and channelized ditches influence sediment pathways by altering hydrologic connectivity, enhancing preferential flow, and affecting in-stream sediment storage (Williams et al. 2016). Here, the 0% and 100% cover crop cases are intentional bounding scenarios, designed to bracket observed adoption levels (8–68%) and explore watershed-scale responses under maximum adoption. Additionally, it can take 10 years to see changes or improvements in soil organic matter, and other aspects of water quality (Meals et al. 2010; Waring et al. 2024; Thapa et al. 2025), although we did not see this in our SWAT results. With cover crops planted on all fields, the model indicated an immediate improvement in water quality. Expanding the use of conservation practices on all agricultural lands can be an important contributor to reducing sediment loads downstream to rivers and reservoirs, as has been found with nutrients (Speir et al. 2022; Aboelnour et al. 2025b).

### TSS Yields Vary Across Watershed Sizes

Yield of TSS may vary between rivers and their tributary streams, but past studies on the relationship between TSS yields and watershed size have shown variable results depending on characteristics of the river basin (Dedkov et al. 2004; Birkinshaw and Bathurst 2006). We predicted that TSS yields would be lower in the larger river basins compared to the small agricultural watersheds, due to the larger drainage area and the potential settling of particles as water velocity and gradient change in rivers. We found that the three river basins generally had lower annual TSS yields than the two small agricultural watersheds. These larger river basins include areas of land uses other than agriculture, such as urban (Tippecanoe = 8%, Kankakee = 8%, Wabash = 9%) and forested areas (Tippecanoe = 11%, Kankakee = 9%, Wabash = 11%), whereas the two small watersheds are dominated by agricultural land use. TSS yields are strongly influenced by land use, where TSS concentrations generally decrease with urban and forest area, and increase with cropland area (Dodds and Whiles 2004; Aboelnour et al. 2025a). Dams and reservoirs within a watershed can also reduce sediment yields (Walling and Fang 2003) by trapping sediment and slowing flow. Additionally, large rivers are further from their headwaters, which can be a dominant source of sediment (Morris and Fan 1997). Here, the ratio of drained area (km^2^) to stream or river length (km) increased from ~2 km in the two small watersheds to 143 km in the Wabash River basin, reflecting that the Wabash drains a much larger area relative to its length. As the depth and slope changed from the two small streams to the larger rivers, both the Reynolds number (Re) and Shields number (*θ*) also changed. We estimated these numbers based on the varying conditions of each waterbody, including velocity, depth, slope, and sediment characteristics (Reynolds 1883; Shields 1936). Reynolds number increased from ~50,000 in the two small streams studied here to ~2,100,000 in the Wabash River, primarily due to the increase in flow velocity and depth, which contributed to a transition to more turbulent flow. However, the Shields number was higher in the small streams, at approximately 0.36, decreasing to around 0.05 in the larger rivers. This change is primarily due to the differences in slope, demonstrating that sediment is more easily mobilized in the small streams (Lamb et al. 2008). Rivers contribute notably to global sediment transport, and have played an important role in shaping landscapes (Ward et al. 2002). While rivers can play a large role in reservoir and downstream sedimentation, our findings highlight that small agricultural watersheds are major contributors of sediment loads to these larger watersheds.

## Conclusions

Overall, we show that sediment yields at the watershed scale are variable, and the effects of cover crops on watershed-scale TSS yields were not always detectable. We observed reduced TSS loss from tile drains draining fields with cover crops planted, and reduced watershed export during high-flow conditions in the watershed with higher cover crop coverage, suggesting that cover crops may limit sediment loss at the field-scale and during periods of elevated flow when fields would otherwise be bare. Our modeling results also showed a decrease in watershed-scale yields when cover crops were planted on 100% of croppable acres, emphasizing the need for conservation in small watersheds, as they are the primary sources of sediment yields compared to large river basins (Dodds and Whiles 2004; Warrick et al. 2015). Advances in sensor technology have allowed for high-frequency measurements of turbidity, and subsequently TSS, thus improving estimates of sediment yields across a range of stream orders (Walling 1994; Bieroza et al. 2023). These advances in monitoring will become increasingly beneficial under changing hydroclimatic conditions. We integrate long-term, high-frequency empirical measurements at both tile drain and watershed scales with scenario-based modeling to show that cover crops can reduce sediment export. By reducing TSS yields at the source, planting winter cover crops can help protect downstream water quality and ecosystem health in the face of a changing climate.

## Supplementary information


Supplementary information


## Data Availability

The data used in this study are publicly available via a GitHub repository and have been archived on Zenodo for long-term access: Pruitt, Abagael (2025), “Pruitt et al. Sediment Paper.” The dataset is available at https://doi.org/10.5281/zenodo.15396886, which corresponds to the GitHub repository at https://github.com/abagaelpruitt/Pruitt-et-al.-Sediment-Paper.
