# Supplementary material for: Quantifying the Impact of Winter Cover Crops on Sediment Export in Small Agricultural Watersheds and Beyond
Source: Environ Manage. 2026 Aug 1;76(8):259. doi: 10.1007/s00267-026-02578-y (PMC13428719; doi:10.1007/s00267-026-02578-y)
Supplement: Supplementary file 1 — Supplementary information [file 267_2026_2578_MOESM1_ESM.docx]

**Quantifying the impact of winter cover crops on sediment export in small agricultural watersheds and beyond**

*Environmental Management*

Abagael N. Pruitt^1,$*^, Jennifer L. Tank^1^, Shannon L. Speir^1,&^, Ursula H. Mahl^1^, Mohamed Aboelnour^2^, Anna E.S. Vincent^1,%^, Todd V. Royer^3^

^1^Department of Biological Sciences, University of Notre Dame, Notre Dame, IN 46556 USA

^2^Environmental Change Initiative, University of Notre Dame, Notre Dame, IN 46556 USA

^3^O’Neill School of Public and Environmental Affairs, Indiana University, Bloomington, IN 47405 USA

^$^Present affiliation: Department of Environmental Toxicology, University of California, Davis, Davis, CA, 95616 USA

^&^Present affiliation: Department of Crop, Soil, and Environmental Science, University of Arkansas, Fayetteville, AR 72701 USA

^%^Present affiliation: Center for Water Research, Northwestern University, Evanston, IL 60208 USA

^*^Corresponding Author: Abagael N. Pruitt, [abagaelpruitt@gmail.com](mailto:abagaelpruitt@gmail.com)

ORCID iDs: ANP <https://orcid.org/0000-0003-1798-4774>; JLT <https://orcid.org/0000-0001-9005-9548>; SLS <https://orcid.org/0000-0002-3624-0327>; UHM <https://orcid.org/0000-0002-1404-1319>; MA <https://orcid.org/0000-0003-2441-1149>; AESV <https://orcid.org/0000-0002-4218-1749>; TVR <https://orcid.org/0000-0002-1945-1560>


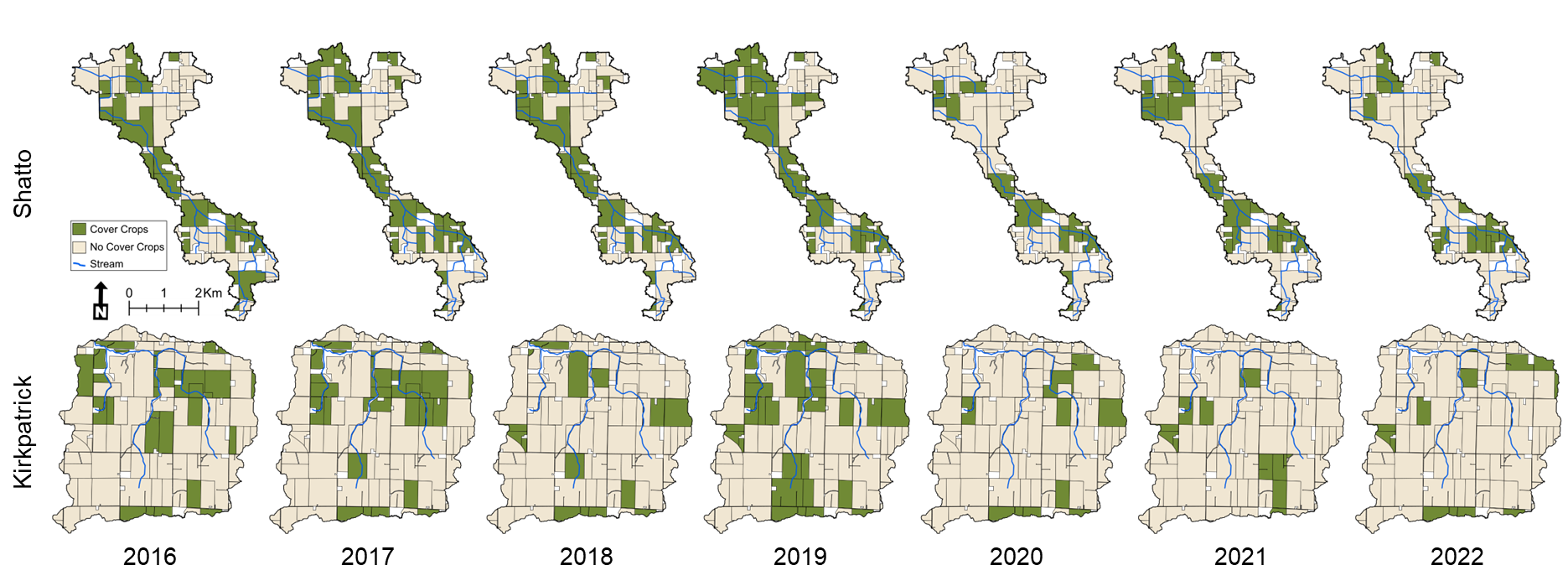


**Figure S1:** Map of cover crop coverage in study watersheds, including Shatto Ditch Watershed (top) and Kirkpatrick Ditch Watershed (bottom) over the seven-water year period. Fields with cover crops planted are in green.
